# ORBIT: a New Paradigm for Genetic Engineering of Mycobacterial Chromosomes

**DOI:** 10.1128/mBio.01467-18

**Published:** 2018-12-11

**Authors:** Kenan C. Murphy, Samantha J. Nelson, Subhalaxmi Nambi, Kadamba Papavinasasundaram, Christina E. Baer, Christopher M. Sassetti

**Affiliations:** a Department of Microbiology and Physiological Systems, University of Massachusetts Medical School, Worcester, Massachusetts, USA; Sequella, Inc.

**Keywords:** *Mycobacterium smegmatis*, bacteriophage genetics, gene replacement, genetic fusions, metabolic engineering, promoter replacements, recombineering, tuberculosis

## Abstract

Two efficient recombination systems were combined to produce a versatile method for chromosomal engineering that obviates the need to prepare double-stranded DNA (dsDNA) recombination substrates. A synthetic “targeting oligonucleotide” is incorporated into the chromosome via homologous recombination mediated by the phage Che9c RecT annealase. This oligonucleotide contains a site-specific recombination site for the directional Bxb1 integrase (Int), which allows the simultaneous integration of a “payload plasmid” that contains a cognate recombination site and a selectable marker. The targeting oligonucleotide and payload plasmid are cotransformed into a RecT- and Int-expressing strain, and drug-resistant homologous recombinants are selected in a single step. A library of reusable target-independent payload plasmids is available to generate gene knockouts, promoter replacements, or C-terminal tags. This new system is called ORBIT (for “oligonucleotide-mediated recombineering followed by Bxb1 integrase targeting”) and is ideally suited for the creation of libraries consisting of large numbers of deletions, insertions, or fusions in a bacterial chromosome. We demonstrate the utility of this “drag and drop” strategy by the construction of insertions or deletions in over 100 genes in Mycobacterium tuberculosis and M. smegmatis.

## Abstract (precis)

We sought to develop a system that could increase the usefulness of oligonucleotide-mediated recombineering of bacterial chromosomes by expanding the types of modifications generated by an oligonucleotide (i.e., insertions and deletions) and by making recombinant formation a selectable event. This paper describes such a system for use in M. smegmatis and M. tuberculosis. By incorporating a single-stranded DNA (ssDNA) version of the phage Bxb1 *attP* site into the oligonucleotide and coelectroporating it with a nonreplicative plasmid that carries an *attB* site and a drug selection marker, we show both formation of a chromosomal *attP* site and integration of the plasmid in a single transformation. No target-specific dsDNA substrates are required. This system will allow investigators studying mycobacterial diseases, including tuberculosis, to easily generate multiple mutants for analysis of virulence factors, identification of new drug targets, and development of new vaccines.

## INTRODUCTION

Following the use of the laborious plasmid cointegration schemes that dominated the early days of gene replacement in bacteria, recent advances in genetic engineering of bacteria have largely relied on phage recombination systems, including both site-specific and homologous systems. For example, it was recognized in 1993 that a nonreplicating plasmid containing the mycobacterial phage L5 *attP* site could be incorporated into the Mycobacterium smegmatis chromosome via integration into the bacterial *attB* site (1); a similar system was described a year later for Escherichia coli (2). Such events are dependent on the expression of phage integrases (Int). Thus, genes expressed from endogenous or regulatable promoters could be delivered into the stable confines of the chromosome in single-copy form, without the physiological artifacts of excessive gene copy numbers or the instability of self-autonomous replication vectors. Site-specific recombination (SSR) systems were the basis for development of the Lambda InCh method for transferring exogenous genes to the E. coli *attB* site (3); development of the series of conditional-replication, integration, and modular (CRIM) plasmids that take advantage of a variety of different phage *attB* sites in E. coli (4); development of mycobacterial phage vectors for delivery of exogenous sequences to mycobacterial chromosomes (5–7); and for many technical modifications of these SSR systems and their substrates for use in metabolic and genetic engineering protocols in a variety of organisms (8–12).

In addition to these site-specific recombination systems, general homologous recombination (HR) systems of phage, such as the Red system of phage lambda, and the RecET systems of Rac prophage and mycobacterial phage Che9c, have been widely used for genetic engineering in a variety of bacteria (13–19; for reviews, see reference 20, 21, and 22). Termed “recombineering,” these procedures have used both double-stranded DNA (dsDNA) and single-stranded DNA oligonucleotides for generating bacterial chromosomal modifications in E. coli and in various bacterial pathogens, including enterohemorrhagic E. coli (23), Salmonella enterica (20, 24–26), Shigella flexneri (27), Pseudomonas aeruginosa (28, 29), Yersinia pestis (30, 31), and M. tuberculosis (18, 19). The Che9c RecT system has been especially useful for the verification of drug-resistant single nucleotide polymorphisms (SNPs) in the search for new drug targets for tuberculosis (32, 33). These general phage recombination systems have also been used for the development of new methodologies for metabolic engineering of industrial microorganisms (34–43). In particular, the Red system annealase, λ Beta protein, has been employed in many protocols for the systematic modification of E. coli. For example, oligonucleotides have been electroporated into cells expressing λ Beta to target the promoter regions of genes, allowing accelerated evolution of E. coli for specific metabolic engineering purposes (44). In concert with these homologous recombination systems, the SSR systems described above have been employed to remove selectable drug cassettes for the construction of markerless gene deletions and fusions (14, 16, 45).

The new system described in this report couples the recombinogenic annealing of an oligonucleotide and the site-specific insertion of a nonreplicating plasmid into a one-step procedure for generating chromosomally tagged genes, deletions, or promoter replacements in M. smegmatis or M. tuberculosis. It takes advantage of a voluminous amount of work done in the laboratory of G. Hatfull on both the Bxb1 phage integration system (5, 46–52) and the Che9 RecET recombineering system (17, 19), without which the development of this methodology would not be possible. It is the first general chromosomal engineering technique that produces a drug-selectable recombinant that does not require the use of either target-specific dsDNA plasmids or PCR-generated recombination substrates. The only target-specific substrate requirement for gene modification is a chemically synthesized oligonucleotide. This “targeting oligo” carries the ssDNA version of the Bxb1 phage *attP* site (48 bases) flanked by 45 to 70 bases of homology to the chromosomal target and is coelectroporated with a nonreplicating “payload plasmid” that contains a Bxb1 *attB* site. The host (M. smegmatis or M. tuberculosis) contains a plasmid that expresses both the Che9c phage RecT annealase and the Bxb1 phage integrase. RecT promotes annealing of the targeting oligonucleotide to the lagging strand template of the replication fork, thus placing the *attP* site into a precise location in the chromosome dictated by the oligonucleotide sequence. In the same outgrowth period, Bxb1 integrase promotes site-specific recombination between the coelectroporated *attB*-containing payload plasmid and the oligonucleotide-derived *attP* site. In this system, the sequence of the oligonucleotide defines the position of the insertion site and the plasmid delivers the payload. For knockouts, the oligonucleotide is designed such that *attP* replaces the target gene. For C-terminal tags, the oligonucleotide is designed to insert *attP* at the end of the coding sequence. In this case, the type of C-terminal tag desired is defined by the selection of the plasmid to be coelectroporated with the oligonucleotide from a library of preexisting payload plasmids. Inherently in this system, a single oligonucleotide designed to create a C-terminal tag can be used with multiple plasmids to create fluorescent, degradation, and epitope-tagged fusions. This new gene modification scheme is called ORBIT (for “oligonucleotide-mediated recombineering followed by Bxb1 integrase targeting”). We describe the use of the system to generate over 100 gene knockouts and fusions in M. smegmatis and M. tuberculosis at high efficiency. This system could easily be employed in other bacteria as well, provided that an ssDNA annealing protein (most likely from an endogenous phage system) is available for use (see Discussion).

## RESULTS

### RecT-promoted oligonucleotide-mediated recombineering—60-bp insertion.

Two different types of recombineering methodologies have been applied to mycobacteria, but neither represents a broadly generalizable approach for genome engineering. Target-specific dsDNA substrates can be used to make diverse and selectable mutations. However, while PCRs with long oligonucleotides can extend homologous regions to lengths of 100 to 150 bp, the most efficient recombinant frequencies are obtained with a selectable marker containing ∼500 bp of flanking homology. When many genes are being interrogated, these dsDNA constructs can be cumbersome to generate for each desired mutation. In contrast, single-stranded oligonucleotides are easily synthesized and can be used to alter one or a few bases of the chromosome at high efficiency. However, these mutations are generally not selectable and are therefore difficult to isolate. An ideal method would leverage easily synthesized and highly efficient oligonucleotide substrates to make selectable mutations. We sought to accomplish this by encoding a phage attachment site (*attP*) in the oligonucleotide that could be used to integrate a selectable marker at a specific chromosomal site via site-specific recombination.

**FIG 1:**
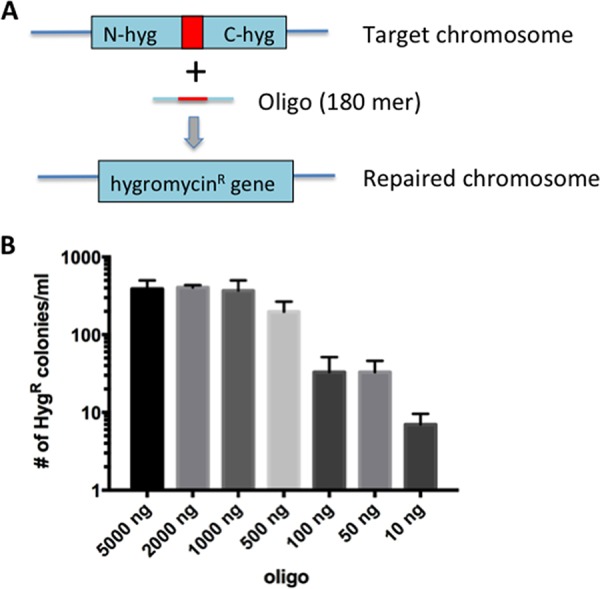
RecT-promoted oligonucleotide-mediated 60-base insertion. (a) Diagram of oligonucleotide-mediated recombineering of a chromosomal target in M. smegmatis. An integrating plasmid (pKM433) at the L5 phage attachment site contains a mutated *hyg* resistance gene due to an internal 60-bp deletion (red square). Electroporation of an oligonucleotide containing the 60 bases missing in the target gene, along with 60 bp of flanking DNA on each side, was electroporated into cells expressing the Che9c RecT function from pKM402. (b) After induction of RecT and preparation of the cells for transformation (as described in Materials and Methods), the cells were electroporated with various amounts of an oligonucleotide (180 mer) that spans the 60-bp deletion of the Hyg resistance cassette in pKM433. Cells were grown overnight in 7H9 broth, and half the culture volume was plated on LB-Hyg plates. The experiment was performed in triplicate; standard errors are shown.

To test the feasibility of this idea, an assay was designed to measure the frequency of incorporation of an oligonucleotide containing an insertion of approximately the size of a 48-bp *attP* site into the mycobacterial chromosome. For this purpose, a hygromycin resistance (Hyg^r^) gene, with an internal 60-bp deletion, was integrated into the L5 phage attachment site of the M. smegmatis chromosome. Oligonucleotides (180-mers) targeting the lagging strand template of the impaired Hyg^r^ marker and containing the missing 60 bases (as well as 60 bases flanking the deletion site) were electroporated into M. smegmatis expressing the Che9c RecT annealase from the anhydrotetracycline (ATc)-inducible Ptet promoter (pKM402) (32). The frequency of oligonucleotide incorporation was determined as the number of Hyg^r^ transformants among the survivors of electroporation (Fig. 1a). The number of Hyg^r^ transformants generated ranged from fewer than 10 to over 300, depending on the amount of oligonucleotide used (Fig. 1b). At about 1 μg of oligonucleotide, the total number of Hyg^r^ recombinants plateaued at approximately 350 transformants/ml, which corresponds to a frequency of 2 × 10^−6^ recombinants per survivor of electroporation. In an experiment where the target contained a 1-bp change creating a premature stop codon in the *hyg* resistance gene, a 60-base oligonucleotide was used to restore Hyg resistance at a frequency of 4 × 10^−4^ recombinants per survivor of electroporation. Thus, the RecT annealase is capable of integrating an oligonucleotide that contains a 60-base insertion into the mycobacterial chromosome, albeit at a frequency which is ∼500-fold lower than that seen with a single base pair change.

### Development of ORBIT.

To convert the recombineering of an oligonucleotide into a selectable event, we determined if coelectroporation of the *attP*-containing oligonucleotide with an *attB*-containing nonreplicating vector (Hyg^r^) into a cell that expresses both the RecT annealase and the phage Bxb1 integrase would allow both homologous and site-specific recombination events to occur within the same outgrowth period. Since the oligonucleotide is designed to direct the integration of the genetic information contained in the nonreplicating plasmid, these elements were termed “targeting oligonucleotide” and “payload plasmid.”

**FIG 2:**
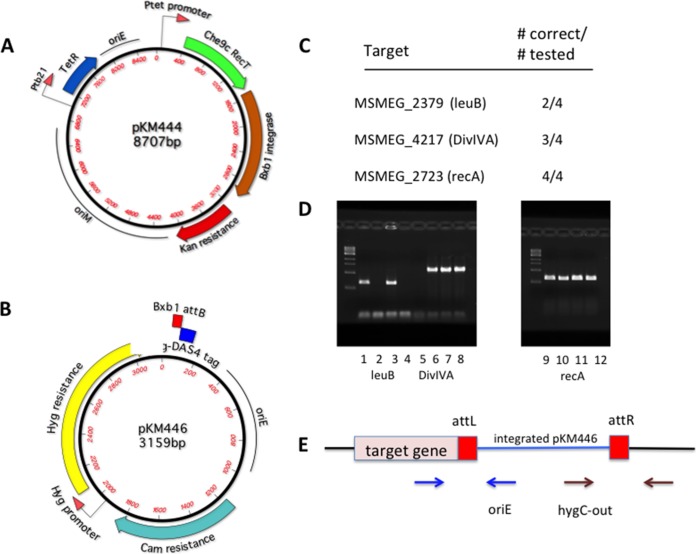
Plasmids constructed for ORBIT. (a) Construct pKM444 expresses the Che9c phage RecT annealase and the Bxb1 phage integrase, both driven from the Ptet promoter. A similar construct (pKM461) additionally contains the *sacRB* counter-selectable marker for curing the plasmid following gene modification. (b) One of the ORBIT payload plasmids (pKM446) used for integration into the chromosomal *attP* site created by an oligonucleotide. In this case, the plasmid payload contains a Flag-DAS+4 inducible degradation tag that is ultimately going to be fused to the 3′ end of the target gene. Cam^r^, chloramphenicol resistance. (c) Three genes in M. smegmatis were targeted for C-terminal tagging. Following the ORBIT protocol for each target gene, the total numbers of colonies obtained (from multiple trials) ranged between 20 to 200 CFU/transformation. Electroporations performed with payload plasmid only (no targeting oligonucleotides) produced, on average, 5-fold-lower total numbers of colonies. The number of correct recombinants (of 4 candidates tested) for each target gene is shown. (d) PCR analysis of the 5′ junctions of each candidate tested. (e) Primer positions for verification by PCR of the recombinants are shown. Blue arrows, 5′ junction; brown arrows, 3′ junction. In each case where a 5′ junction was verified, the 3′ junction was also verified (not shown). The 5′ junctions were confirmed by DNA sequencing.

Two plasmids were generated to test this methodology. pKM444 produces the recombination functions. This mycobacterial shuttle vector expresses both the Che9c phage RecT annealase and the Bxb1 phage integrase (Int) from an anhydrotetracycline (ATc)-inducible Ptet promoter (Fig. 2a). pKM446 is a payload plasmid that does not replicate in mycobacteria. This vector includes a *hyg* resistance marker for selection in mycobacteria and a Bxb1 *attB* site (Fig. 2b). Adjacent to the *attB* site is a sequence encoding both a Flag tag and a DAS+4 peptide tag designed to be in frame with a targeted chromosomal gene following integration of the plasmid. The DAS+4 tag directs a fusion protein for degradation via the ClpXP system upon expression of the SspB adapter protein (53, 54). Targeting oligonucleotides were designed to direct the integration of *attP* to the 3′ ends of the M. smegmatis *recA*, *divIVA*, and *leuB* genes, immediately before the stop codon. A site-specific recombination event between the inserted *attP* and the coelectroporated pKM446 would generate a DAS+4 fusion to these target genes (see Table S1 in the supplemental material for the list of oligonucleotides used in this study). Targeting oligonucleotides, which anneal to the lagging strand template of the replication fork, were coelectroporated with the pKM446 payload plasmid into M. smegmatis expressing both Che9c RecT annealase and Bxb1 integrase. Among the Hyg^r^ colonies resulting from these transformations, 9 of 12 candidates tested by PCR contained the expected recombinant structure, in which pKM446 was inserted between *attR* and *attL* sites at the predicted oligonucleotide-directed integration site (Fig. 2c to e). The fusion of the target genes to the Flag-DAS+4 degradation tag was verified by sequencing the PCR products of the 5′ junctions. The design of the ORBIT oligonucleotide used to create the *recA*–Flag-DAS+4 fusion is described in Fig. S1 in the supplemental material; the relevant sequence of the pKM446-integrating plasmid is shown in Fig. S2, and the sequence of a generic chromosomal Flag-DAS+4 fusion in shown in Fig. S3.

10.1128/mBio.01467-18.1 FIG S1 Design of the ORBIT oligonucleotide. The Bxb1 *attP* sequence is placed between the last codon and the stop codon of the M. smegmatis *recA* gene. The *attP* site (brown) is flanked by 70 bases of *recA* C-terminal sequence (blue) and 70 bases of downstream sequence (black), including the *recA* stop codon (red). Given the rules for selecting the lagging strand for efficient recombineering (see Fig. S4), the reverse complement of the sequence shown here was used as the ORBIT oligonucleotide. Download FIG S1, TIF file, 3.0 MB. Copyright © 2018 Murphy et al. 2018 Murphy et al. This content is distributed under the terms of the Creative Commons Attribution 4.0 International license.

10.1128/mBio.01467-18.2 FIG S2 ORBIT integrating plasmid pKM446. The integrating pKM446 plasmid contains an *attB* site, the Flag tag, and the DAS+4 tag. A “CG” base pair is inserted between *attB* and the Flag tag to ensure that the tags are in frame with the target gene following integration of the plasmid. For this to occur, the ORBIT oligonucleotide has to be designed as described in the Fig. S1 legend. Download FIG S2, TIF file, 1.5 MB. Copyright © 2018 Murphy et al. 2018 Murphy et al. This content is distributed under the terms of the Creative Commons Attribution 4.0 International license.

10.1128/mBio.01467-18.3 FIG S3 Chromosomal layout of an ORBIT-promoted Flag-DAS+4 fusion. The sequence shown contains the last codon of the target gene (NNN), the 43-bp *attL* site (orange-brown) created by a crossover between *attP* and *attB* (the crossover core sequence is underlined) and a CG base pair (violet), which was included in the ORBIT integrating plasmid to fuse the 43-bp *attL* site to both the Flag tag (green) and the DAS+4 tag (blue). Finally, a double stop codon for the chromosomal fusion is supplied by the payload plasmid (red). Note that, as required, the *attL* site lacks a stop codon. Download FIG S3, TIF file, 1.5 MB. Copyright © 2018 Murphy et al. 2018 Murphy et al. This content is distributed under the terms of the Creative Commons Attribution 4.0 International license.

10.1128/mBio.01467-18.5 TABLE S1 ORBIT oligonucleotides for M. tuberculosis target genes and M. smegmatis target genes. Download Table S1, XLSX file, 0.05 MB. Copyright © 2018 Murphy et al. 2018 Murphy et al. This content is distributed under the terms of the Creative Commons Attribution 4.0 International license.

To test the functionality of the tag and to verify that the mutated locus represented the only copy of the targeted gene in the cell, we took advantage of the DAS+4 sequence, which promotes degradation of the tagged proteins upon induction of SspB in M. smegmatis *and* M. tuberculosis (55–57). Two different *sspB* expression systems were used, both of which induce degradation of the target protein either by adding ATc (Tet-OFF) or by removing ATc (Tet-ON).

**FIG 3:**
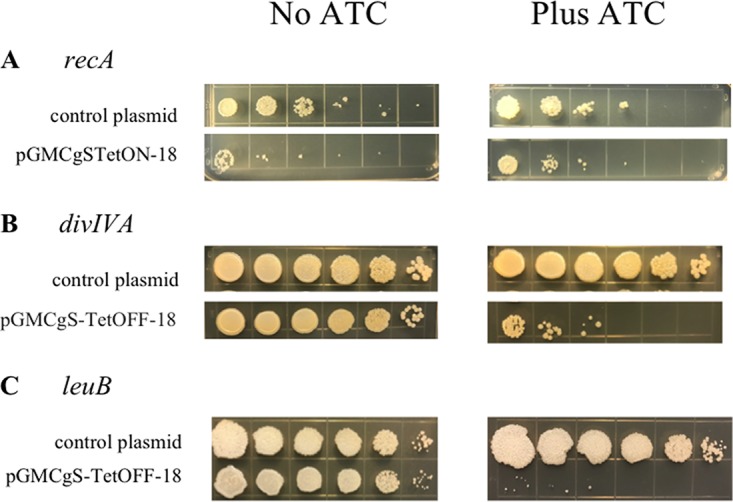
Knockdown phenotypes of ORBIT-generated DAS+4-tagged strains. The growth phenotypes of the Flag-DAS+4 tagged strains constructed as described in the Fig. 2 legend were analyzed after transformation of an SspB-expressing plasmid. (a) The *recA*–Flag-DAS+4 strain was transformed with an SspB-producing plasmid, pGMCgS-TetON-18 (streptomycin resistance [Strep^r^]), under the control of the reverse TetR repressor. In this scenario, RecA is degraded in the absence of anhydrotetracycline (ATc). Ten-fold serial dilutions of the cells grown overnight without ATc were spotted on LB-streptomycin plates, either with or without ATc, and the cells were exposed to 20 J/m^2^ of UV. Preferential UV sensitivity of the *recA*–Flag-DAS+4 strain in the absence of ATc was determined by comparing the levels of growth seen with and without the inducer. (b) The *divIVA*–Flag-DAS+4 strain was transformed with an SspB-producing plasmid, pGMCgS-TetOFF-18 (Strep^r^), under the control of the wild-type TetR repressor. In this case, DivIVA is expected to be depleted in the presence of ATc. Preferential growth of the *DivIVA*–Flag-DAS+4 strain on LB plates in the presence of ATc was determined by comparing the levels of growth seen with and without the inducer. (c) The experiment was performed as described for panel B, except that *leuB* was the target and the cells were plated on 7H10-AD plates.

Cells containing the *recA*-DAS+4 fusion were transformed with pGMCgS-TetON-18, allowing the induction of SspB in the absence of ATc; RecA function was then quantified using a UV resistance assay. Grown in the presence of ATc, the *recA*-DAS+4-tagged strain containing the SspB-expressing plasmid and a strain containing a control plasmid showed nearly identical levels of UV sensitivity (Fig. 3a, right). In contrast, the sensitivity of the tagged strain with SspB was enhanced in the absence of ATc (Fig. 3a, left). The *divIVA* and *leuB* DAS+4-tagged strains were transformed with pGMCgS-TetOFF-18, which produces SspB upon ATc addition. In both cases, addition of ATc inhibited the growth of these mutants, consistent with the essentiality of these genes on the media used in this study (Fig. 3b and c). The defect of LeuB depletion could be reversed by the addition of leucine to the plates, verifying that that this phenotype was linked to the engineered mutation (data not shown). Thus, the ORBIT method could generate function-altering mutations without the need to construct either target-specific plasmids or long dsDNA recombineering substrates.

**FIG 4:**
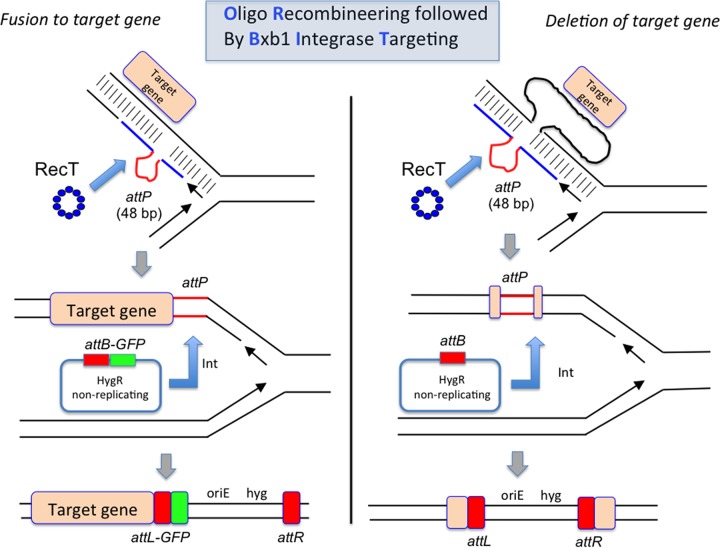
ORBIT-promoted gene alteration. The ORBIT process is initiated at the replication fork. An oligomer containing a single-stranded version of the Bxb1 *attP* site (top pictures, red lines) is coelectroporated with an *attB*-containing nonreplicating plasmid into a mycobacterial host cell expressing both RecT annealase and Bxb1 integrase. RecT promotes annealing of the oligonucleotide to the lagging strand template. Following DNA replication through this region, an *attP* site is formed in the chromosome (middle pictures). In the same outgrowth period, Bxb1 integrase promotes site-specific insertion of the plasmid into the chromosome (*attB* × *attP*). (Left side) The oligonucleotide is designed such that *attP* is inserted just before the stop codon. The integration event fuses the GFP tag in frame to the 3′ end of the target gene (with an *attL* site in frame between them); the recombinant is selected for by Hyg^r^. (Right side) The oligonucleotide is designed such that *attP* replaces the target gene and the plasmid integration event allows hygromycin resistance to be used to select for the knockout.

The scheme of using RecT and Bxb1 integrase simultaneously to promote modification of a chromosomal target gene is diagrammed in Fig. 4; the process is called ORBIT (for “oligonucleotide-mediated recombineering followed by Bxb1 integrase targeting”). In theory, targeting oligonucleotides could also be designed to delete a portion of the chromosome during the ORBIT reaction (Fig. 4, right side) to create knockouts. Demonstrations of such ORBIT-promoted knockouts are described below.

### Parameters of ORBIT-promoted gene targeting.

**FIG 5:**
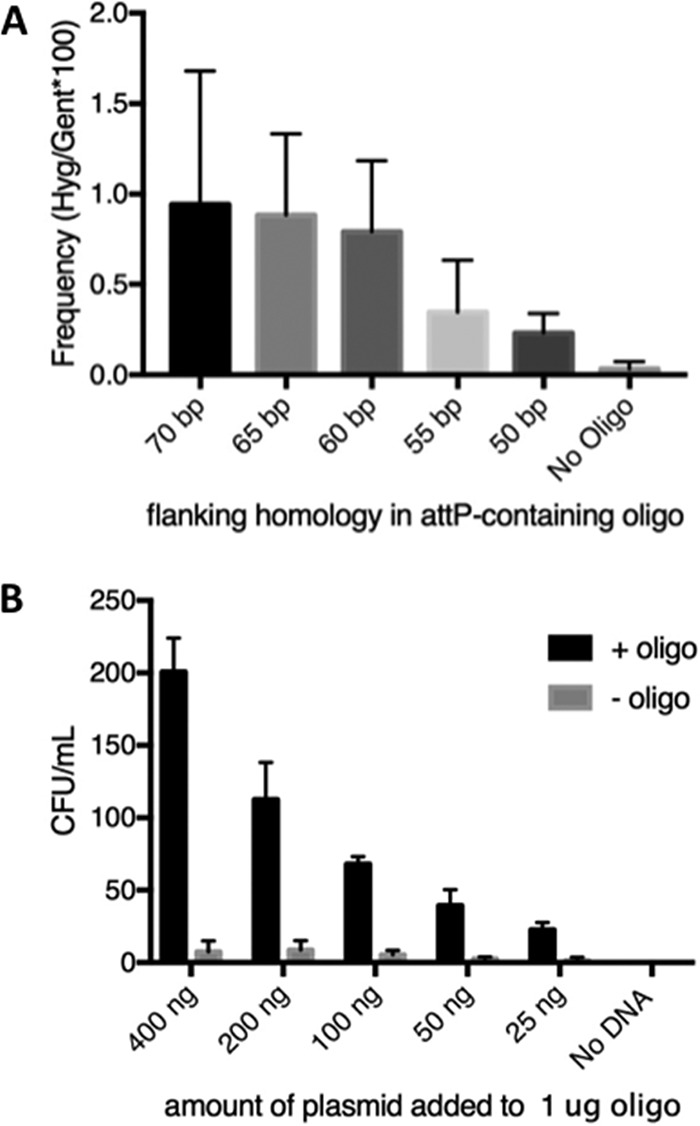
Parameters of the ORBIT process. (A) The amount of target homology flanking the *attP* site in an oligonucleotide designed to create a *polA*–Flag-DAS+4 fusion in M. smegmatis was examined as a function of recombinant formation (Hyg^r^). One microgram of each oligonucleotide was electroporated with 200 ng of pKM446. The frequency of targeting is expressed as the percentage of Hyg^r^ transformants following integration of pKM446 relative to a transformation control (20 ng of gentamicin resistance [Gen^r^] plasmid pKM390). Experiments were performed in triplicate; standard errors are shown. Gent, gentamicin. (B) Colony counts were determined after electroporation of 1 μg of an oligonucleotide with 70-base flanks (designed to create a *polA*–Flag-DAS+4 fusion) with various amounts of pKM446. CFU counts per milliliter were determined following overnight growth of the electroporation mixtures in 2 ml LB. Experiments were performed in triplicate; standard errors are shown.

Two features of ORBIT that were optimized were the length of the homologous arms (HAs) in the targeting oligonucleotide and the relative amounts of oligonucleotide and nonreplicating plasmid used for coelectroporation. The HAs of a *polA*-targeting oligonucleotide were adjusted to lengths of 50 to 70 bases (oligonucleotide lengths, including *attP*, were 148 to 188 bases in length). In each case, the *attP* sequence was placed between the last codon of *polA* and its stop codon (similarly to the *recA* oligonucleotide used as described above; see Fig. S1). The oligonucleotides were mixed with 200 ng of payload plasmid pKM446 and transformed into M. smegmatis containing pKM444 induced with ATc; a gentamicin-resistant integrating plasmid was included as a transformation control. While there was variability with respect to the number of Hyg-resistant transformants from each electroporation, oligonucleotides containing longer HAs produced more transformants (Fig. 5a). For HAs with sequences below 40 bp in length, no Hyg^r^ transformants were observed above the number seen in the no-oligonucleotide control (data not shown).

We also examined the optimal ratio of oligonucleotide to plasmid. Targeting oligonucleotide (1 μg) was coelectroporated with various amounts of the pKM446 payload plasmid. More Hyg^r^ transformants were observed when more plasmid was used, but there was also a small increase in the number of oligonucleotide-independent transformants that presumably represent illegitimate recombinants (Fig. 5b). PCR screening confirmed that 39/40 Hyg^r^ transformants recovered in this experiment (using 8 candidates of each transformation) represented the desired oligonucleotide-directed recombination events. As a rule, we generally combined 1 μg of oligonucleotide with 200 ng of plasmid in the transformations described below.

### ORBIT-promoted knockdowns and knockouts in M. smegmatis *and* M. tuberculosis.

**FIG 6:**
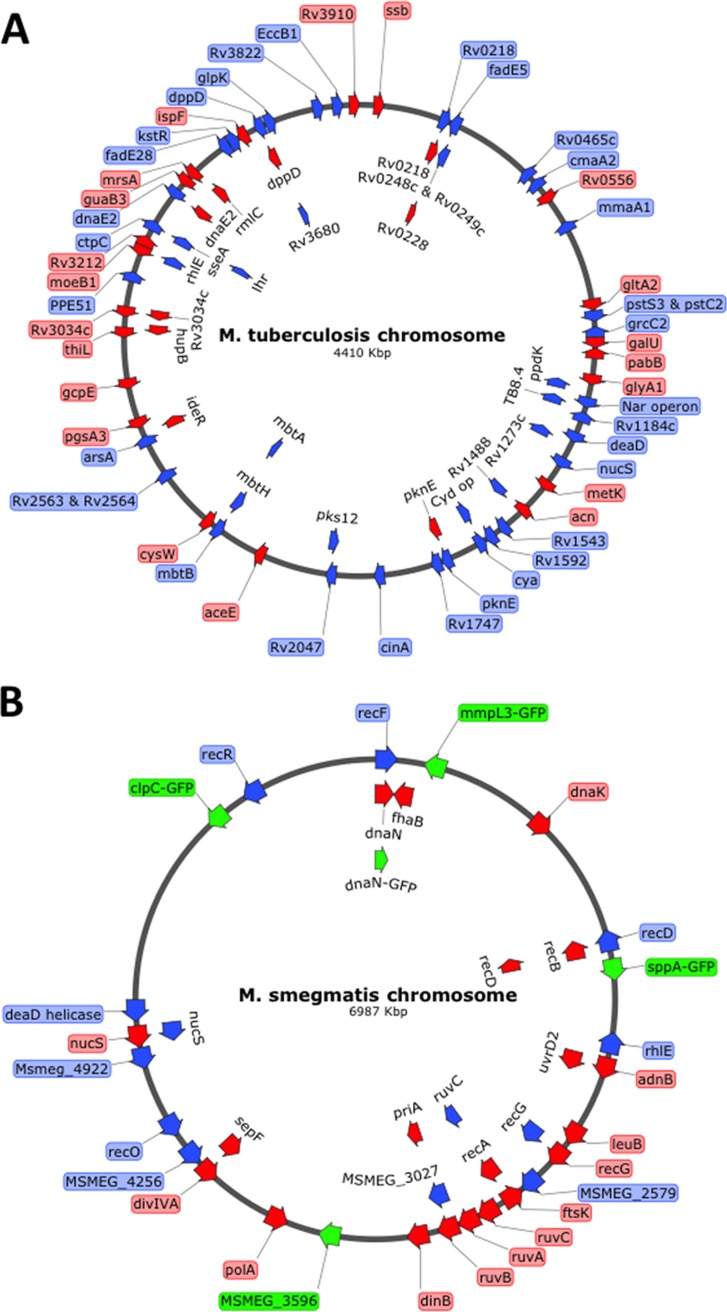
ORBIT-generated insertions and deletions in M. tuberculosis and M. smegmatis. Gene deletions and modifications were performed at a variety of positions in the chromosomes of both M. tuberculosis (a) and M. smegmatis (b) using ORBIT. In most cases, the oligonucleotides contained an *attP* site flanked by 70 bases of target homology. Insertions (C-terminal DAS+4 or His-Flag tags) are shown in red, deletions are shown in blue, and GFP tags are shown in green. Descriptions of all the modifications performed by ORBIT are provided in Table 1 (for M. smegmatis) and Table 2 (for M. tuberculosis).

To determine if ORBIT-promoted modifications could be generally engineered throughout the chromosome, we targeted a variety of genes in M. tuberculosis and M. smegmatis. For C-terminal tags, the *attP* site was placed between the last translating codon of the target gene and its stop codon (see Fig. S1 for how *attP* is placed within the M. smegmatis *recA* gene to create a C-terminal fusion). The ORBIT system is designed such that all oligonucleotides used for any type of C-terminal fusion are constructed in a similar manner (i.e., with the *attP* site placed as indicated in Fig. S1). This is important, as all ORBIT-integrating plasmids that are designed to generate C-terminal fusions have a 2-bp sequence (CG) placed between the *attB* site and the relevant tag (see Fig. S2). These 2 bp, together with the 43 bp of the *attL* site created after integration, ensure that all target genes are translationally fused to the tag supplied by the integrating vector (see Fig. S3 for details). For knockouts, the *attP* site was flanked by 60 to 70 bases, which typically included the first and last 10 codons of the target gene (including the start and termination codons), which is expected to result in the deletion of intervening chromosomal sequence. Overall, we have made over 100 strains where the target genes were either deleted or C-terminally tagged (see Fig. 6 and Tables 1 and 2). For most of these targets, between 5 to 50 colonies typically arose after plating 25% of the overnight outgrowth (0.5 ml), though in some cases, plates contained over 100 colonies. Usually, only 2 to 4 Hyg^r^ candidates were analyzed by PCR to identify at least one strain that contained the payload plasmid integrated into the site designated by the targeting oligonucleotide. Most of the targeting oligonucleotides used for these genomic modifications contained either 60 or 70 bases of flanking homology. An oligonucleotide targeting the *aceE* gene containing only 45 bases of homology on each side of *attP* also produced the desired recombinant in 4 of 6 clones. However, lowering flanking homologies to below 40 bases decreased the percentage of correct recombinants dramatically, largely as a result of an increased number of illegitimate recombination events.

**TABLE 1:** ORBIT-promoted *M. smegmatis* modifications

Gene	Designation	Function	No. of correct recombinants/total no. tested
Flag-Das4 tags			
*leuB*	MSMEG_2379	3-Isopropylmalate dehydrogenase	2/4
*recA*	MSMEG_2723	Recombinase	4/4
*divIVA*	MSMEG_4217	DivIVA protein	3/4
*dnaN*	MSMEG_0001	DNA polymerase III, beta subunit	2/2
*dinB*	MSMEG_3172	DNA gyrase	2/2
*dnaK*	MSMEG_0709	Chaperone (heat shock)	1/2
*recD*	MSMEG_1325	ExoV, α-subunit	2/4
*recB*	MSMEG_1327	ExoV, β-subunit	2/2
*adnB*	MSMEG_1943	ATP-dependent helicase/recombinase	2/2
*recG*	MSMEG_2403	ATP-dependent DNA helicase	2/2
*ftsK*	MSMEG_2690	DNA translocase	3/6
*ruvA*	MSMEG_2944	Holliday junction branch migration	1/2
*ruvB*	MSMEG_2945	Holliday junction branch migration	3/4
*ruvC*	MSMEG_2943	Holliday junction resolvase	1/2
*priA*	MSMEG_3061	Replication restart	2/2
*polA*	MSMEG_3839	DNA polymerase I	4/6
*dinB*	MSMEG_3172	DNA polymerase IV	2/2
*fhaB*	MSMEG_0034	FHA domain-containing protein	6/12
*sepF*	MSMEG_4219	Interaction with FtsZ and MurG	1/2
*uvrD2*	MSMEG_1952	ATP-dependent DNA helicase	2/2
*nucS*	MSMEG_4493	ssDNA-binding protein	3/4

GFP tags			
*dnaN*	MSMEG_0001	DNA polymerase III, beta subunit	nr^*a*^
*mmpL3*	MSMEG_0205	MmpL family protein	nr
*sppA*	MSMEG_1476	Signal peptide peptidase	nr
*clpC*	MSMEG_6091	ATP-dependent protease ATP-binding protein	nr
	MSMEG_3596	ATPase	nr

Deletions			
Δ*recD*	MSMEG_1325	ExoV, α-subunit	2/4
Δ*recF*	MSMEG_0003	Replication repair protein	2/4
Δ*recG*	MSMEG_2403	ATP-dependent DNA helicase	2/2
Δ*recO*	MSMEG_4491	DNA repair protein	1/2
Δ*ruvC*	MSMEG_2943	Holliday junction resolvase	2/6
Δ*recR*	MSMEG_6279	Recombination protein	4/6
Δ*nusS*	MSMEG_4923	Mismatch repair function	nr
	MSMEG_4922	Conserved hypothetical protein	nr
Δ*rhlE*	MSMEG_1930	RNA helicase	nr
Δ*deaD*	MSMEG_5042	*deaD* RNA helicase	nr
	MSMEG_2579	Unknown	nr
	MSMEG_3027	Unknown	nr
	MSMEG_4256	NLP/P60 family protein	nr

a nr, not reported.

**TABLE 2:** ORBIT-promoted *M. tuberculosis* modifications

Rv no.	Gene	Function
Knockouts		
Rv0244c	*fadE5*	Probable acyl-CoA^*a*^ dehydrogenase FadE5
Rv0248c	Rv0248c	Probable succinate dehydrogenase
Rv0249c	Rv0249c	Probable succinate dehydrogenase
Rv0465c	Rv0465c	Probable transcriptional regulatory protein
Rv0503c	*cmaA2*	Cyclopropane-fatty-acyl-phospholipid synthase
Rv0645c	*mmaA1*	Methoxy mycolic acid synthase
Rv0928	*pstS3*	Periplasmic phosphate-binding lipoprotein PstS3
Rv0929	*pstC2*	Phosphate transport integral membrane ABC transporter
Rv0989c	*grcC2*	Probable polyprenyl-diphosphate synthase
Rv1161–Rv1164	NarG-NarI	Nitrate reduction
Rv1174c	TB8.4	Low-molecular-weight T-cell antigen TB8.4
Rv1184c	Rv1184c	Possible exported protein
Rv1253	*deaD*	Probable cold-shock DeaD-box protein A homolog
Rv1273c	Rv1273c	Probable drug transport transmembrane ABC transporter
Rv1321	*nucS*	Probable mismatch repair protein
Rv1488	Rv1488	Possible exported conserved protein
Rv1543	Rv1543	Possible fatty acyl-CoA reductase
Rv1592	Rv1592	Conserved hypothetical protein
Rv1620c–Rv1623c	*cyd* operon	Respiratory chain
Rv1621c	*cydD*	Transmembrane ATP-binding protein ABC transporter CydD
Rv1623c	*cydA*	Probable integral membrane cytochrome *d* ubiquinol oxidase
Rv1625c	*cya*	adenylate cyclase
Rv1747	Rv1747	Probable conserved transmembrane ABC transporter
Rv1901	*cinA*	Probable CinA-like protein CinA
Rv2047	Rv2047	Conserved hypothetical protein
Rv2048c	Pks12	Polyketide synthase
Rv2383c	*mbtB*	Phenyloxazoline synthetase
Rv2384	*mbtA*	Salicyl-AMP ligase (SAL-AMP ligase) + salicyl–S-ArCP synthetase
Rv2563	Rv2563	Glutamine transport transmembrane protein ABC transporter
Rv2564	Rv2564	Glutamine transport ATP-binding protein ABC transporter
Rv2684	*arsA*	Probable arsenic transport integral membrane protein ArsA
Rv3136	PPE51	PPE family protein PPE51
Rv3211	*rhiE*	Probable ATP-dependent RNA helicase
Rv3270	*ctpC*	Probable metal cation-transporting P-type ATPase C
Rv3283	*sseA*	Probable thiosulfate sulfurtransferase SseA
Rv3296	*lhr*	Probable ATP-dependent helicase Lhr (large-helicase-related protein)
Rv3544c	*fadE28*	Probable acyl-CoA dehydrogenase
Rv3574	*kstR*	Transcriptional regulatory protein
Rv3680	Rv3680	Probable anion transporter ATPase
Rv3696	*glpK*	Probable glycerol kinase
Rv3822	Rv3822	Conserved hypothetical protein
Rv3869	*eccB1*	ESX-1 type VII secretion system protein

Insertions (Flag-Das4 tags)		
Rv0054	*ssb*	Single-strand-binding protein
Rv0218	Rv0218	Probable conserved transmembrane protein
Rv0228	Rv0228	Probable integral membrane acyltransferase
Rv0556	Rv0556	Probable conserved transmembrane protein
Rv0896	*gltA2*	Probable citrate synthase
Rv0993	*galU*	UTP–glucose-1-phosphate uridylyltransferase GalU
Rv1005c	*pabB*	Probable para-aminobenzoate synthase component
Rv1093	*glyA1*	Serine hydroxymethyltransferase
Rv1392	*metK*	Probable S-adenosylmethionine synthetase
Rv1475c	*acn*	Probable iron-regulated aconitate hydratase
Rv1743	*pknE*	Probable transmembrane serine/threonine protein kinase E
Rv2241	*aceE*	Pyruvate dehydrogenase E1 component
Rv2398c	*cysW*	Probable sulfate transport membrane protein ABC transporter
Rv2746c	*pgsA3*	Probable PGP synthase PgsA3
Rv2868c	*gcpE*	Probable GcpE protein
Rv2977c	*thiL*	Probable thiamine-monophosphate kinase
RV2986c	*hupB*	DNA-binding protein HU homolog
Rv3034c	Rv3034c	Possible transferase
Rv3034c	Rv3034c	Possible transferase
Rv3206c	*moeB1*	Probable molybdenum cofactor biosynthesis protein MoeB1
Rv3212		Conserved alanine-valine-rich protein
Rv3370c	*dnaE2*	DNA polymerase III (alpha chain)
Rv3410c	*guaB3*	Probable inosine-5′-monophosphate dehydrogenase
Rv3441c	*mrsA*	Probable phospho-sugar mutase
Rv3465	*rmlC*	dTDP-4-dehydrorhamnose 3,5-epimerase
Rv3484	*cpsA*	Possible conserved protein CpsA
Rv3581c	*ispF*	Probable 2C-methyl-d-erythritol 2,4-cyclodiphosphate synthase
Rv3663c	*dppD*	Probable dipeptide transport ATP-binding protein
Rv3910	Rv3910	Probable conserved transmembrane protein

a CoA, coenzyme A.

In order to cure recombinants of the RecT-Int-producing plasmid following modification, pKM461, a *sacB*-containing derivative of pKM444, was constructed. A number of genes were tagged in a pKM461-bearing strain (including M. smegmatis *recG*, *dnaN*, and *ftsK*) and were then plated directly on Hyg-sucrose plates both to select for the recombinant and to cure the strain of the RecT-Int producer. This process appears to produce moderately fewer colonies than had been observed previously with pKM444 but can more rapidly produce plasmid-free mutants.

Genetic modifications with ORBIT should be stable. An excision event promoted by the Bxb1 SSR system requires an additional factor (called gp47 [49]) which is not present in the hosts used for these experiments. The stability of ORBIT-mediated modifications was verified by observing that the pKM468 integrated payload plasmid, after integration into the M. smegmatis MSMEG_3596 and MSMEG_0250 genes, was not lost after more than 100 generations of growth in the absence of drug selection, as shown by detection of equal numbers of colonies on LB plates with and without ATc. In addition, both PCR analysis and fluorescence microscopy were employed to verify the continued presence of the green fluorescent protein (GFP) tag in both strains.

All the ORBIT-promoted gene modifications described in Tables 1 and 2 were done with M. smegmatis or M. tuberculosis already transformed with either pKM444 or pKM461. In order to investigate the possibility that transient expression of RecT and Bxb1 integrase would be sufficient to promote recombinant formation by ORBIT, we deleted the oriM sequence from pKM444 and electroporated the resulting plasmid with pKM446 and an ORBIT oligonucleotide designed to fuse the Flag-DAS+4 tag to the C-terminal end of *dnaN*. In standard ORBIT experiments, this target typically gave the highest number of colonies relative to other M. smegmatis genes, possibly as a result of its close proximity to the chromosomal origin of replication. However, in experiments performed using the pKM444 derivative missing oriM, the numbers of colonies observed were no greater than the numbers of colonies observed with cells that were transformed with the plasmids but not with the oligonucleotide. Furthermore, results of PCR analyses of eight candidates from this experiment were negative for the presence of the 5′ junction of the expected recombinant. It seems likely that RecT is required to be present in abundance prior to entry of the oligonucleotide into the cell to protect the oligonucleotide from digestion by cellular nucleases and/or to efficiently deliver the oligonucleotide to the replication fork.

### Expanding the library of ORBIT-mediated modifications.

In order to expand the types of modifications that can be made via ORBIT, we built a set of payload plasmids containing the Bxb1 *attB* site fused to different types of tags. In addition to the Flag-DAS+4 plasmids, we generated plasmids to create C-terminal targeted fusions with combinations of enhanced GFP (eGFP), mVenus, SNAP, CLIP, Myc and His epitopes and tobacco etch virus (TEV) cleavage sites. These tags facilitate both protein localization studies performed by fluorescence analysis and affinity purifications (Table 3). In addition, we created plasmids to generate chromosomal knockouts and promoter replacements using either hygromycin resistance or zeocin resistance (Zeo^r^) as a method of selection for the recombinant (see Table 3). Any number of payload plasmids can be matched with a single targeting oligonucleotide to generate a variety of functional gene modifications.

**TABLE 3:** ORBIT integration plasmids^*a*^

Plasmid name	Type of modification	Drug resistance marker	Addgene ID
C-terminal tags			
pKM446	C-terminal tag: Flag-DAS tag	Hyg^r^	108321
pKM468	C-terminal tag: EGFP-4×Gly-TEV–Flag-6×His	Hyg^r^	108434
pKM469	C-terminal tag: Venus-4×Gly-TEV–Flag-6×His	Hyg^r^	108435
pKM489	C terminal tag: SNAP tag	Hyg^r^	108437
pKM490	C-terminal tag: CLIP tag	Hyg^r^	109281
pKM491	C-terminal tag: 4×Gly-TEV–Flag-6×His	Hyg^r^	109282
pKM492	C-terminal tag: 4×Gly-TEV-Myc-6×His	Hyg^r^	109283
pKM493	C-terminal tag: TEV–Flag-4×Gly-EGFP	Hyg^r^	In process
pKM495	C-terminal tag: Flag-DAS tag	Zeo^r^	109284

Knockouts			
pKM464	Knockout	Hyg^r^	108322
pKM496	Knockout	Zeo^r^	109301

Promoter replacements			
pKM464	Replacement of endogenous promoter with P_Hyg_	Hyg^r^	108322
pKM496	Replacement of endogenous promoter with P_GroEL_ (op-rbs)^*b*^	Zeo^r^	109301
pKM508	Replacement of endogenous promoter with P21 (op-rbs)	Zeo^r^	In process
pKM509	Replacement of endogenous promoter with P38 (op-rbs)	Zeo^r^	In process

a All plasmids contain the chloramphenicol resistant cassette (Cam^r^) for use during isolation of the plasmid grown in *E. coli*. ID, identifier; In process, to be added at a later date.

b Optimized ribosome binding site (AGAAAGGAGGAAGGA).

As with any chromosomal engineering method, one must consider the effect of the modification on expression of downstream functions, especially if the target gene is within an operon. The pKM464 ORBIT knockout plasmid was designed such that once integrated, the *hyg* gene is positioned at the junction of the insertion site, allowing downstream genes to be transcribed by the *hyg* promoter. In our experience, this is generally sufficient for the generation of most mutations (see Table 1 for knockouts generated in M. smegmatis). For additional options, pKM495 (for Flag-DAS+4 tags) and pKM496 (for knockouts) contain an additional promoter (P_GroEL_) to drive higher transcription levels downstream of the insertion site (Table 3). Similar plasmids can be generated with promoters of various strengths, as needed.

### GFP fusions.

**FIG 7:**
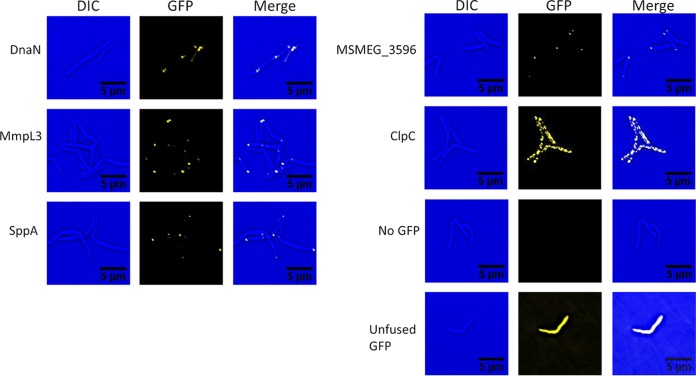
ORBIT-generated GFP fusions. M. smegmatis cells containing GFP-tagged target genes were grown in 7H9-AD-Tween 80 to an optical density of 0.8. One microliter of the culture was spotted on an agarose pad for microscopy. Each bacterial strain was imaged using differential interference contrast (DIC) and GFP channels, as described in Material and Methods.

To demonstrate the functionality of these additional tags, ORBIT was used to construct eGFP fusions with a number of M. smegmatis genes. We tagged DnaN (the beta subunit of DNA polymerase III) and MmpL3 (the essential mycolate transporter suspected to reside at the pole), two proteins known to be located in discrete cytoplasmic foci, and three genes with unknown distribution patterns. The pKM468 eGFP payload plasmid (Table 3) was used to tag each gene *in situ* at the endogenous chromosomal locus. MmpL3-eGFP and DnaN-eGFP were concentrated at the predicted localization site of each protein (Fig. 7). For DnaN-GFP, the punctate spots identify positions of the replication forks that occurred in nonpolar regions of the cell (see Fig. 7), in accordance with previous observations (58). eGFP-tagged alleles of MSMEG_3596, SppA, and ClpC were found at distinct sites, a result which differed from the diffuse cytosolic distribution of unfused eGFP. Overall, ORBIT is a rapid and efficient method to modify native genes in the chromosome with functionally distinct tags without having to create target- and modification-specific dsDNA recombination substrates. Also, by expressing each gene at its native level, aberrant localization or complex formation due to overexpression can be avoided.

### Promoter replacements.

One can also integrate *attB*-containing ORBIT plasmids to change the endogenous levels of expression of a chromosomal gene. We constructed a series of ORBIT plasmids containing promoters of different strengths that can be used to drive the expression of genes downstream of the plasmid insertion site (see the promoter replacement plasmids listed in Table 3). To use these plasmids, the ORBIT oligonucleotide is designed to replace the endogenous promoter with *attP*. The target gene’s ribosome-binding site, if recognized, can also be deleted, replaced, or left intact, depending on the final level of expression desired. To test this scheme, we performed ORBIT in an M. smegmatis strain where a chromosomal *lacZ* gene is under the control of the mycobacterial PAg85 promoter containing a weak ribosome-binding site.

**FIG 8:**
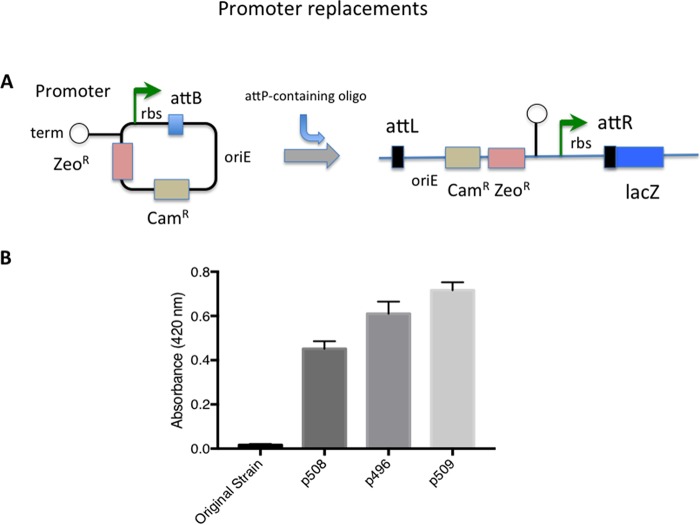
Promoter replacements. (a) Diagram of ORBIT-generated promoter replacements. In the nonreplicating plasmid, the promoter to be inserted into the chromosome is placed to the left of the *attB* site. A *TrrnB* terminator is placed upstream of this promoter to prevent read-through from the plasmid backbone. The oligonucleotide is designed to place *attP* just upstream of the target gene in place of the endogenous promoter. Following integration, the promoter and inserted ribosome-binding site drive expression of the chromosomal target gene (*lacZ*). (b) ORBIT was carried out with plasmids pKM496 (P_GroEL_), pKM508 (P_imyc_), and pKM509 (P38) with an oligonucleotide that deletes the endogenous promoter. Extracts of the cells were made, and beta-galactosidase assays were performed in triplicate (standard error bars are shown). The higher amounts of beta-galactosidase present in the engineered strains, relative to the starting strain, were likely due to the presence of the optimized ribosome-binding site following each promoter.

ORBIT was used to replace PAg85 with one of three different promoters: Pimyc, P_GroEL_, or P38. Plasmids containing these promoters are listed according to increasing strength of expression (D. Schnappinger, personal communication). The promoters were transferred to ORBIT integration plasmids and placed upstream of the Bxb1 *attB* site (see Fig. 8a). An optimized ribosome-binding site (rbs: AGAAAGGAGGAAGGA) was included between the promoters and the *attB* site to increase the overall level of expression of β-galactosidase relative to the starting strain, where an endogenous Shine-Delgarno sequence could not be recognized. In this arrangement, the final recombinant had an *attR* site situated between the promoter-SD sequence and the translational start site of the target gene (see Fig. 8a). (Alternatively, an altered SD sequence can be included in the oligonucleotide sequence, thereby placing it closer to the start codon of the target gene.) ORBIT recombinants were identified by resistance to zeocin and verified by chromosomal PCR analysis as described above. The total amount of β-galactosidase in each extract increased in accordance with the expected strengths of the three promoters used in these assays (Fig. 8b). Thus, endogenous promoters can be easily replaced to modify the expression level of a chromosomally located target gene.

### Markerless gene deletions.

The ORBIT protocol represents a unique way to construct markerless gene deletions in a process involving reversal of plasmid integration step. The Bxb1 phage produces the gp47 protein, which has been shown to be a recombination directionality factor (RDF) (49). Along with the Bxb1 integrase, gp47 acts to promote site-specific recombination between *attL* and *attR*, restoring the *attP* site. By replacing *recT* with *gp47* in pKM461 and exchanging the Hyg^r^ gene with a Zeo^r^ cassette (*ble*), a plasmid was generated that expresses both gp47 and integrase under the control of Ptet (pKM512). The M. smegmatis ORBIT-generated deletion strain ΔMSMEG_4392, not yet cured of the Kan^r^ plasmid pKM461, was transformed with pKM512 (Zeo^r^), grown in the presence of anhydrotetracycline (ATc), and plated on 7H10-sucrose plates as described in Materials and Methods. Of 37 colonies of strain ΔMSMEG_4392 treated in this manner, 7 were sensitive to hygromycin; in addition, all of them were sensitive to both kanamycin and zeocin. Site-specific recombination between the *attR* and *attL* sites replaces the deleted portion of MSMEG_4392 with the *attP* site, creating an in-frame deletion, which was verified by sequencing in 4 of the 7 Hyg^S^ colonies described above. Thus, mutants of mycobacteria devoid of any antibiotic resistance can be generated following construction of ORBIT-generated deletions.

## DISCUSSION

The λ Red recombineering system has been critical for genome engineering of E. coli and related bacteria. While λ Red does not function well in bacteria more distantly related to E. coli, endogenous phage recombination systems have been identified and employed to promote recombineering in these organisms. One of the more notable examples of a phage recombination system utilized in this way is the Che9c mycobacterial RecET system, identified by Van Kessel and Hatfull and used for recombineering of the M. smegmatis and M. tuberculosis chromosomes (18, 19, 22). This recombineering system has been used to construct numerous knockouts and modifications of mycobacterial targets (22, 32, 59, 60). However, unlike λ Red recombineering in E. coli, PCR substrates of 50-bp flanking homology do not work efficiently for recombineering in M. smegmatis and M. tuberculosis, though longer homologies can be added by PCRs performed with long oligonucleotides containing 50 to 100 bp of additional target homologies (61). To increase recombination efficiency further, one needs to construct recombineering substrates containing ∼500 bp of flanking homology for each specific modification. Similarly, specialized transduction also requires the laborious construction and packaging of a different recombination substrate-containing phagemid for each genome modification (62).

ORBIT overcomes the limitations of existing methods by combining components of two different efficient recombination systems from mycobacteria: the Che9c annealase from the general homologous recombination system and the Bxb1 integrase from the site-specific recombination system. The construction of long dsDNA recombination substrates is replaced by the synthesis of a targeting oligonucleotide, and the availability of a library of payload plasmids allows a single oligonucleotide to be used to generate a wide variety of functional modifications. The ability to direct the insertion of functionally distinct payloads creates a precise and versatile “drag and drop” strategy for genome engineering.

The utility of ORBIT extends beyond the simple generation of mutants. Since targeting oligonucleotides are easily generated, the system is well suited for the construction of libraries of mutants. The targeting oligonucleotide loop containing the 48-bp *attP* site can be extended to 60 bp (Fig. 1) to include a nearly infinite number of unique DNA barcode sequences, which would allow each mutant in a large ORBIT-generated pool to be monitored independently by PCR or next-generation sequencing. The variety of modifications that can be made with the existing collection of payload plasmids opens new avenues for functional screening of mutant libraries that are generated using this method. ORBIT can also be used for genome reduction strategies in M. tuberculosis. The largest deletion generated in this study was the one-step 12-kb deletion of the *pks12* gene, and it is likely that larger deletions are possible. Finally, though most of the payload plasmids described in this report were designed to modify endogenous genes, one could also use this strategy to place exogenous elements, such as large clusters of biosynthetic genes, into any specified position in the mycobacterial chromosome.

The paradigm of ORBIT is likely to be useful in many different bacterial species. The Bxb1 integrase requires no host functions to carry out the site-specific and directional (*attB* × *attP*) recombination reaction. It is for these reasons that Bxb1 integrase has been selectively employed for genetic manipulations in both bacterial and mammalian cells (63, 64). Thus, the key to the development of ORBIT for other microbial systems would be to find a λ Beta or RecT-like annealase that promotes some level of oligonucleotide-mediated recombineering. Datta et al. (65) have tested a number of single-stranded annealing proteins (SSAPs) from both Gram-positive and Gram-negative bacteria for oligonucleotide-mediated recombineering in E. coli. While the recombineering efficiency ranged over 3 orders of magnitude, some SSAPs from distant species worked as well as the λ Beta protein in E. coli, suggesting that some annealases are easily transferable between species. An annealase appropriate for use in ORBIT would have to be able to promote annealing of an oligonucleotide containing a 48-bp *attP* insertion. Note, however, that even if oligonucleotide recombineering were to occur at low frequencies (∼10^−6^) with such a substrate, the number of *attP* sites generated could very well be adequate for the Bxb1 integrase to promote integration of a nonreplicating plasmid, thus making integration of the oligonucleotide a selectable event.

## MATERIALS AND METHODS

### Bacterial strains.

The M. smegmatis strains used in this study were derived from mc^2^155; the M. tuberculosis strains were all derived from H37Rv.

### Media.

M. smegmatis was grown in Middlebrook 7H9 broth with 0.05% Tween 80, 0.2% glycerol, 0.5% bovine serum albumin (BSA), 0.2% dextrose, and 0.085% NaCl; transformants were selected on LB plates (Difco) with appropriate drugs. M. tuberculosis was grown in 7H9 broth with 0.05% Tween 80, 0.2% glycerol, and OADC (oleic acid-albumin-dextrose-catalase; Becton, Dickinson); transformants were selected on 7H10 plates with 0.5% glycerol and OADC. When needed, the following antibiotics (concentrations) were added: kanamycin (20 μg/ml), streptomycin (20 μg/ml), hygromycin (50 μg/ml), and zeocin (25 μg/ml).

### Plasmids.

Plasmids containing the P_imyc_ promoter (66), the P_GroEL_ promoter, and P38 promoter were obtained from D. Schnappinger and S. Ehrt, as were plasmids pGMCgS-TetOFF-18 and pGMCgS-TetON-18, where the E. coli SspB adapter protein is under the control of the wild-type and reverse TetR repressors, respectively. Plasmids constructed for this study are described in Tables 3 and 4 and are available at the Addgene plasmid repository site. Details of plasmid constructions are available upon request.

**TABLE 4:** ORBIT-testing and RecT-Int-expressing plasmids

Plasmid	Functions	Drug resistance	Addgene ID
pKM433	Phage L5 integrating vector; HygΔ60-bp internal deletion; oriE	Zeo^r^	
pKM444	P_Tet_-Che9c RecT-Bxb1 Int; TetR, oriE, oriM	Kan^r^	108319
pKM461	P_Tet_-Che9c RecT-Bxb1 Int; SacRB; TetR, oriE, oriM	Kan^r^	108320
pKM512	P_Tet_-Bxb1 GP47-Int; SacRB; TetR, oriE, oriM	Zeo^r^	In process

### Oligonucleotides.

Oligonucleotides used for ORBIT were obtained from IDT as Ultramers at a concentration of 100 uM and delivered in 96-well plates; they were supplied desalted with no further purification. Oligonucleotides were diluted 10-fold in 10 mM Tris-HCl (pH 8.0), and final concentrations (250 to 350 ng/ml) were determined by analysis of absorbance maxima at 260 nm (Abs_260_) using a conversion factor of an optical density (OD) of 1 = 20 μg/ml oligonucleotide. ORBIT plasmids (200 ng) were mixed with 1 μg of oligonucleotide prior to electroporation, unless stated otherwise in figure legends.

### Design of the ORBIT oligonucleotide.

The sequence of oligonucleotides flanking the *attP* site used for ORBIT must be derived from the lagging strand of the replication fork. For design of an ORBIT oligonucleotide, a dsDNA sequence file of a target gene was created, starting 200 bp upstream of the initiation codon and ending 200 bp downstream of the stop codon. The Bxb1 *attP* site (shown in Fig. S4 in the supplemental material) was inserted into the target sequence file for the type of modification required (i.e., knockout, C-terminal tag, or promoter replacement) as described in Results. For selection of the lagging strand, the rules are as follows: if the transcriptional direction of the target gene is pointing toward the chromosomal origin in either replicore (green arrows in Fig. S4), then select the top strand (5′ to 3′) of the “target sequence + attP” file as the lagging strand DNA in the oligonucleotide. If the transcriptional direction of the target gene is pointing away from the chromosomal origin in either replicore (red arrows in Fig. S4), then select the bottom strand (5′ to 3′) of the “target sequence + attP” file as the lagging strand in the oligonucleotide. The example shown in Fig. S4 is for M. smegmatis. Apply the same rules for M. tuberculosis, but assume that the *dif* region occurs at 2.2 Mb. For further details, see reference 22.

10.1128/mBio.01467-18.4 FIG S4 Design of the ORBIT oligonucleotide. To identify the lagging strand from a sequence file of a target gene (reading 5′ to 3′ from the start codon), the *attP* site is first inserted into the desired position. The ORBIT oligonucleotide sequence will correspond to the top strand if the target gene is transcribed toward the *ori* sequence (see, e.g., the green arrows) or to the bottom strand if the target gene is transcribed toward the *dif* region (see, e.g., the red arrows). Download FIG S4, TIF file, 1.5 MB. Copyright © 2018 Murphy et al. 2018 Murphy et al. This content is distributed under the terms of the Creative Commons Attribution 4.0 International license.

### ORBIT electroporations.

A culture of M. smegmatis containing plasmid pKM444 (or pKM461) was started overnight by adding 100 to 150 μl of a fresh saturated stock culture to 20 ml of 7H9 media containing 20 μg/ml kanamycin in a 125-ml flask. Cells were grown on a swirling platform at 37°C. The next day, at an OD (600 nm) of 0.5, anhydrotetracycline (ATc) was added to reach a final concentration of 500 ng/ml. The culture was placed back on the swirling platform at 37°C for 2.5 to 3 h until the culture OD was ∼1.0. The culture was then placed on ice (with swirling) for 10 min and centrifuged in 50-ml conical tubes at 4,000 rpm for 10 min in a chilled centrifuge. The supernatant was removed, and the pellet was gently resuspended in 2 ml of 10% cold glycerol and then brought up to 20 ml with 10% cold glycerol. The tube was inverted a few times to promote mixing. The centrifugation and washing steps were repeated. After the second wash, the cells were collected by centrifugation and resuspended in 2 ml of 10% cold glycerol. Aliquots (380 μl) of electrocompetent cells were added to sterile Eppendorf tubes containing 1 μg of an *attP*-containing oligonucleotide and 200 ng of an *attB*-containing plasmid (except where noted otherwise in figure legends). The cells and DNA were mixed by pipetting and transferred to ice-cooled electroporation cuvettes (0.2 cm path length). The cells were shocked with an electroporator at settings of 2.5 kV, 1,000 Ω, and 25 μF. Following electroporation, the cells were resuspended in 2 ml 7H9 media and rolled at 37°C overnight. The following day, two 0.5-ml portions of the culture were spread on LB plates containing 50 μg/ml hygromycin (or 25 μg/ml zeocin). Plates were incubated for 4 to 5 days at 37°C, and recombinant candidate colonies were picked and suspended in 2 ml 7H9 media containing 50 μg/ml hygromycin (or 25 μg/ml zeocin) and grown overnight at 37°C. Control electroporations with plasmid but without oligonucleotides were also performed.

Electroporations with M. tuberculosis were performed in a similar manner, with the following modifications. Cells containing pKM444 (or pKM461) were grown in 30 ml 7H9 media containing OADC, 0.2% glycerol, 0.05% Tween 80, and 20 μg/ml kanamycin. At an OD of ∼0.8, ATc was added to the culture to reach a final concentration of 500 ng/ml. After ∼8 h of swirling at 37°C, 3 ml of 2 M glycine was added to the culture. The cells were shaken at 37°C overnight (16 h to 20 h in total following induction), collected by centrifugation, and processed as described above, except that all steps were performed at room temperature. Recombinant candidate colonies were picked after 3 weeks into 5 ml 7H9-OADC-Tween containing 50 μg/ml hygromycin and grown with shaking for 4 to 5 days at 37°C.

### PCR analysis for verification of recombinants.

Recombinants were verified by PCR analysis; *Taq* polymerase was obtained from Denville Scientific, Inc. PCRs were performed with 30-μl volumes and contained 125 μM deoxynucleoside triphosphates (dNTPs), 5% dimethyl sulfoxide (DSMO), 1 μM primers, 2 μl of an M. smegmatis overnight culture (or of a heat-inactivated M. tuberculosis culture), and 0.2 μl of *Taq* polymerase. M. tuberculosis cells (OD around 1.5) were heat inactivated at 85°C for 50 min prior to removal from the biosafety level 3 (BSL3) laboratory. The PCR program consisted of an initial step of 95°C for 5 min (to lyse the cells); 30 cycles of 30 s at 95°C, 30 s at 58°C, and 1 min at 72°C; and a final polymerization step of 5 min at 72°C. Correctly sized PCR fragments were generated from both junctions of the payload plasmid inserted into the chromosome (see Fig. 2e). In each case, the 5′ junction was verified by the use of a target-specific primer and an “oriE” primer (CCTGGTATCTTTATAGTCCTGTCG); the 3′ junction was verified by the use of a target-specific primer and a “HygC-out” primer (TGCACGGGACCAACACCTTCGTGG or GAGGAACTGGCGCAGTTCCTCTGG). In some cases, the 5′ junction PCR was verified by sequencing. Target-specific primers contained sequences at least 100 bp upstream (5′) and downstream (3′) of the chromosomal sequences flanking the *attP* site in the ORBIT oligonucleotide. For knockouts, an additional PCR was performed to verify the absence of the target gene in the recombinant.

### Spot titer assays.

Following ORBIT-promoted C-terminal tagging of M. smegmatis *recA*, *divIVA*, and *leuB* genes, cells were transformed with either pGMCgS-TetOFF-18 or pGMCgS-TetON-18 (SspB-producing plasmids) by selection for streptomycin resistance (20 μg/ml); ORBIT plasmid pKM444 (Kan^r^) was not cured beforehand. Cells were grown overnight in 7H9-AD-Tween broth containing 20 μg/ml streptomycin. Ten-fold serial dilutions of the cultures were made in phosphate-bufferd saline (PBS)-Tween, and 10-μl volumes of the dilutions were spotted on LB or 7H10-AD-Tween plates containing 20 μg/ml streptomycin, either with or without 500 ng/ml anhydrotetracycline. Plates were wrapped in aluminum foil and incubated for 3 to 4 days at 37°C. For the *recA* strains, plates were exposed to UV prior to incubation. See the Fig. 3 legend for additional details.

### Fluorescence microscopy.

Bacterial cells were mounted on 1% agar pads and imaged with a DeltaVision Personal DV microscope followed by deconvolution using SoftwoRx software (Applied Precision). Further processing was performed using FIJI software (67). Image brightness and contrast were adjusted for visibility, and the files were converted to 600 dots per inch (dpi). Representative cells are shown from multiple images of each strain.

### Beta-galactosidase activity assay.

A 5-ml volume of culture at an OD of 0.8 to 1.0 was pelleted and resuspended in 1 ml of freshly prepared Z buffer (50 mM Na_2_HPO_4_ [pH 7.0], 10 mM KCl, 1 mM MgSO_4_, 50 mM β-mercaptoethanol). Cells were lysed by bead beating 4 times at 6.5 m/s for 30 s followed by centrifugation for 10 min to harvest the supernatant. Protein concentrations were measured with a NanoDrop instrument. For the activity assay, 10-μg volumes of protein and Z buffer (total volume of 100 μl) were added in triplicate to a 96-well microplate. The reaction was started with 20 μl of 4 mg/ml *o*-nitrophenyl-β-d-galactopyranoside (ONPG) mixed with sodium phosphate buffer (0.1 M; pH 7.0). Once sufficient yellow color had developed, the reaction was terminated with 50 μl of 1 M sodium carbonate. Final absorbance of the sample was measured at 420 nm in a plate reader.

### Markerless gene deletions.

The M. smegmatis ORBIT-generated deletion strain ΔSMEG_4392 harboring the pKM464 plasmid (Hyg^r^) was transformed with pKM512 (Zeo^r^), a plasmid expressing the Bxb1 excisionase functions (gp47 and Int) and SacRB. The selection for zeocin resistance allows curing of pKM461 (Kan^r^) by plasmid incompatibility. After overnight growth of the Zeo^r^ transformant in 7H10 containing 500 ng/ml anhydrotetracycline, cells were plated on 7H10 plates containing 10% sucrose. Single colonies from these plates were streaked on 7H10, 7H10-Hyg, and 7H10-Zeo plates. Colonies found sensitive to hygromycin were also sensitive to both kanamycin and zeocin.

## References

[1] Lee MH, Hatfull GF 1993 Mycobacteriophage L5 integrase-mediated site-specific integration in vitro. J Bacteriol 175:6836–6841. doi:10.1128/jb.175.21.6836-6841.1993. 8226625 PMC206807

[2] Hasan N, Koob M, Szybalsk W 1994 *Escherichia coli* genome targeting, I. Cre-lox-mediated in vitro generation of ori^−^ plasmids and their in vivo chromosomal integration and retrieval. Gene 150:51–56. doi:10.1016/0378-1119(94)90856-7. 7959062

[3] Boyd D, Weiss DS, Chen JC, Beckwith J 2000 Towards single-copy gene expression systems making gene cloning physiologically relevant: lambda InCh, a simple *Escherichia coli* plasmid-chromosome shuttle system. J Bacteriol 182:842–847. doi:10.1128/JB.182.3.842-847.2000. 10633125 PMC94354

[4] Haldimann A, Wanner BL 2001 Conditional-replication, integration, excision, and retrieval plasmid-host systems for gene structure-function studies of bacteria. J Bacteriol 183:6384–6393. doi:10.1128/JB.183.21.6384-6393.2001. 11591683 PMC100134

[5] Kim AI, Ghosh P, Aaron MA, Bibb LA, Jain S, Hatfull GF 2003 Mycobacteriophage Bxb1 integrates into the Mycobacterium smegmatis groEL1 gene. Mol Microbiol 50:463–473. doi:10.1046/j.1365-2958.2003.03723.x. 14617171

[6] Morris P, Marinelli LJ, Jacobs-Sera D, Hendrix RW, Hatfull GF 2008 Genomic characterization of mycobacteriophage Giles: evidence for phage acquisition of host DNA by illegitimate recombination. J Bacteriol 190:2172–2182. doi:10.1128/JB.01657-07. 18178732 PMC2258872

[7] Pham TT, Jacobs-Sera D, Pedulla ML, Hendrix RW, Hatfull GF 2007 Comparative genomic analysis of mycobacteriophage Tweety: evolutionary insights and construction of compatible site-specific integration vectors for mycobacteria. Microbiology 153:2711–2723. 17660435 10.1099/mic.0.2007/008904-0 PMC2884959

[8] Gaj T, Sirk SJ, Barbas CF III. 2014 Expanding the scope of site-specific recombinases for genetic and metabolic engineering. Biotechnol Bioeng 111:1–15. doi:10.1002/bit.25096. 23982993 PMC4097888

[9] Krappmann S 2014 Genetic surgery in fungi: employing site-specific recombinases for genome manipulation. Appl Microbiol Biotechnol 98:1971–1982. doi:10.1007/s00253-013-5480-y. 24407452

[10] Nkrumah LJ, Muhle RA, Moura PA, Ghosh P, Hatfull GF, Jacobs WR Jr, Fidock DA 2006 Efficient site-specific integration in *Plasmodium falciparum* chromosomes mediated by mycobacteriophage Bxb1 integrase. Nat Methods 3:615–621. doi:10.1038/nmeth904. 16862136 PMC2943413

[11] Olorunniji FJ, Rosser SJ, Stark WM 2016 Site-specific recombinases: molecular machines for the genetic revolution. Biochem J 473:673–684. doi:10.1042/BJ20151112. 26965385

[12] Srivastava V, Thomson J 2016 Gene stacking by recombinases. Plant Biotechnol J 14:471–482. doi:10.1111/pbi.12459. 26332944 PMC11389045

[13] Murphy KC 1998 Use of bacteriophage lambda recombination functions to promote gene replacement in Escherichia coli. J Bacteriol 180:2063–2071. 9555887 10.1128/jb.180.8.2063-2071.1998 PMC107131

[14] Zhang Y, Buchholz F, Muyrers JP, Stewart AF 1998 A new logic for DNA engineering using recombination in *Escherichia coli*. Nat Genet 20:123–128. doi:10.1038/2417. 9771703

[15] Yu D, Ellis HM, Lee EC, Jenkins NA, Copeland NG, Court DL 2000 An efficient recombination system for chromosome engineering in *Escherichia coli*. Proc Natl Acad Sci U S A 97:5978–5983. doi:10.1073/pnas.100127597. 10811905 PMC18544

[16] Datsenko KA, Wanner BL 2000 One-step inactivation of chromosomal genes in *Escherichia coli* K-12 using PCR products. Proc Natl Acad Sci U S A 97:6640–6645. doi:10.1073/pnas.120163297. 10829079 PMC18686

[17] Marinelli LJ, Hatfull GF, Piuri M 2012 Recombineering: a powerful tool for modification of bacteriophage genomes. Bacteriophage 2:5–14. doi:10.4161/bact.18778. 22666652 PMC3357384

[18] van Kessel JC, Hatfull GF 2007 Recombineering in *Mycobacterium tuberculosis*. Nat Methods 4:147–152. doi:10.1038/nmeth996. 17179933

[19] van Kessel JC, Hatfull GF 2008 Efficient point mutagenesis in mycobacteria using single-stranded DNA recombineering: characterization of antimycobacterial drug targets. Mol Microbiol 67:1094–1107. doi:10.1111/j.1365-2958.2008.06109.x. 18221264

[20] Sawitzke JA, Thomason LC, Costantino N, Bubunenko M, Datta S, Court DL 2007 Recombineering: in vivo genetic engineering in *E. coli*, *S. enterica*, and beyond. Methods Enzymol 421:171–199. doi:10.1016/S0076-6879(06)21015-2. 17352923

[21] Murphy KC 11 1 2016, posting date. λ recombination and recombineering. EcoSal Plus 2016. doi:10.1128/ecosalplus.ESP-0011-2015. PMC11575712 27223821

[22] Murphy KC, Papavinasasundaram K, Sassetti CM 2015 Mycobacterial recombineering. Methods Mol Biol 1285:177–199. doi:10.1007/978-1-4939-2450-9_10. 25779316

[23] Murphy KC, Campellone KG 2003 Lambda Red-mediated recombinogenic engineering of enterohemorrhagic and enteropathogenic E. coli. BMC Mol Biol 4:11. doi:10.1186/1471-2199-4-11. 14672541 PMC317293

[24] Czarniak F, Hensel M 2015 Red-mediated recombineering of Salmonella enterica genomes. Methods Mol Biol 1225:63–79. doi:10.1007/978-1-4939-1625-2_4. 25253248

[25] Nasvall J, Knoppel A, Andersson DI 2017 Duplication-insertion recombineering: a fast and scar-free method for efficient transfer of multiple mutations in bacteria. Nucleic Acids Res 45:e33. doi:10.1093/nar/gkw1078. 27899661 PMC5389514

[26] Uzzau S, Figueroa-Bossi N, Rubino S, Bossi L 2001 Epitope tagging of chromosomal genes in Salmonella. Proc Natl Acad Sci U S A 98:15264–15269. doi:10.1073/pnas.261348198. 11742086 PMC65018

[27] Ranallo RT, Barnoy S, Thakkar S, Urick T, Venkatesan MM 2006 Developing live Shigella vaccines using lambda Red recombineering. FEMS Immunol Med Microbiol 47:462–469. doi:10.1111/j.1574-695X.2006.00118.x. 16872384

[28] Lesic B, Rahme LG 2008 Use of the lambda Red recombinase system to rapidly generate mutants in *Pseudomonas aeruginosa*. BMC Mol Biol 9:20. doi:10.1186/1471-2199-9-20. 18248677 PMC2287187

[29] Liang R, Liu J 2010 Scarless and sequential gene modification in Pseudomonas using PCR product flanked by short homology regions. BMC Microbiol 10:209. doi:10.1186/1471-2180-10-209. 20682065 PMC2924854

[30] Derbise A, Lesic B, Dacheux D, Ghigo JM, Carniel E 2003 A rapid and simple method for inactivating chromosomal genes in Yersinia. FEMS Immunol Med Microbiol 38:113–116. doi:10.1016/S0928-8244(03)00181-0. 13129645

[31] Sun W, Wang S, Curtiss R. III, 2008 Highly efficient method for introducing successive multiple scarless gene deletions and markerless gene insertions into the *Yersinia pestis* chromosome. Appl Environ Microbiol 74:4241–4245. doi:10.1128/AEM.00940-08. 18487404 PMC2446500

[32] Ioerger TR, O’Malley T, Liao R, Guinn KM, Hickey MJ, Mohaideen N, Murphy KC, Boshoff HIM, Mizrahi V, Rubin EJ, Sassetti CM, Barry CE, Sherman DR, Parish T, Sacchettini JC 2013 Identification of new drug targets and resistance mechanisms in *Mycobacterium tuberculosis*. PLoS One 8:e75245. doi:10.1371/journal.pone.0075245. 24086479 PMC3781026

[33] Singh V, Dhar N, Pató J, Kolly GS, Korduláková J, Forbak M, Evans JC, Székely R, Rybniker J, Palčeková Z, Zemanová J, Santi I, Signorino-Gelo F, Rodrigues L, Vocat A, Covarrubias AS, Rengifo MG, Johnsson K, Mowbray S, Buechler J, Delorme V, Brodin P, Knott GW, Aínsa JA, Warner DF, Kéri G, Mikušová K, McKinney JD, Cole ST, Mizrahi V, Hartkoorn RC 2017 Identification of aminopyrimidine-sulfonamides as potent modulators of Wag31-mediated cell elongation in mycobacteria. Mol Microbiol 103:13–25. doi:10.1111/mmi.13535. 27677649

[34] Kolisnychenko V, Plunkett G III, Herring CD, Feher T, Posfai J, Blattner FR, Posfai G 2002 Engineering a reduced *Escherichia coli* genome. Genome Res 12:640–647. doi:10.1101/gr.217202. 11932248 PMC187512

[35] Alper H, Fischer C, Nevoigt E, Stephanopoulos G 2005 Tuning genetic control through promoter engineering. Proc Natl Acad Sci U S A 102:12678–12683. doi:10.1073/pnas.0504604102. 16123130 PMC1200280

[36] Gross F, Ring MW, Perlova O, Fu J, Schneider S, Gerth K, Kuhlmann S, Stewart AF, Zhang Y, Muller R 2006 Metabolic engineering of *Pseudomonas putida* for methylmalonyl-CoA biosynthesis to enable complex heterologous secondary metabolite formation. Chem Biol 13:1253–1264. doi:10.1016/j.chembiol.2006.09.014. 17185221

[37] Miao V, Coëffet-Le Gal M-F, Nguyen K, Brian P, Penn J, Whiting A, Steele J, Kau D, Martin S, Ford R, Gibson T, Bouchard M, Wrigley SK, Baltz RH 2006 Genetic engineering in *Streptomyces roseosporus* to produce hybrid lipopeptide antibiotics. Chem Biol 13:269–276. doi:10.1016/j.chembiol.2005.12.012. 16638532

[38] Murphy KC 2009 Chromosomal engineering strategies, chapter 7, p 7-1 to 7-28. In Smolke CD (ed), The metabolic pathway engineering handbook: tools and applications. CRC Press—Taylor and Francis Group, Boca Raton, FL.

[39] Hu S, Fu J, Huang F, Ding X, Stewart AF, Xia L, Zhang Y 2014 Genome engineering of *Agrobacterium tumefaciens* using the lambda Red recombination system. Appl Microbiol Biotechnol 98:2165–2172. doi:10.1007/s00253-013-5412-x. 24297480

[40] Gust B, Challis GL, Fowler K, Kieser T, Chater KF 2003 PCR-targeted Streptomyces gene replacement identifies a protein domain needed for biosynthesis of the sesquiterpene soil odor geosmin. Proc Natl Acad Sci U S A 100:1541–1546. doi:10.1073/pnas.0337542100. 12563033 PMC149868

[41] Wenzel SC, Gross F, Zhang Y, Fu J, Stewart AF, Muller R 2005 Heterologous expression of a myxobacterial natural products assembly line in pseudomonads via red/ET recombineering. Chem Biol 12:349–356. doi:10.1016/j.chembiol.2004.12.012. 15797219

[42] Fu J, Wenzel SC, Perlova O, Wang J, Gross F, Tang Z, Yin Y, Stewart AF, Muller R, Zhang Y 2008 Efficient transfer of two large secondary metabolite pathway gene clusters into heterologous hosts by transposition. Nucleic Acids Res 36:e113. doi:10.1093/nar/gkn499. 18701643 PMC2553598

[43] Katashkina JI, Hara Y, Golubeva LI, Andreeva IG, Kuvaeva TM, Mashko SV 2009 Use of the lambda Red-recombineering method for genetic engineering of *Pantoea ananatis*. BMC Mol Biol 10:34. doi:10.1186/1471-2199-10-34. 19389224 PMC2682490

[44] Wang HH, Isaacs FJ, Carr PA, Sun ZZ, Xu G, Forest CR, Church GM 2009 Programming cells by multiplex genome engineering and accelerated evolution. Nature 460:894–898. doi:10.1038/nature08187. 19633652 PMC4590770

[45] Cherepanov PP, Wackernagel W 1995 Gene disruption in *Escherichia coli*: TcR and KmR cassettes with the option of Flp-catalyzed excision of the antibiotic-resistance determinant. Gene 158:9–14. doi:10.1016/0378-1119(95)00193-A. 7789817

[46] Mediavilla J, Jain S, Kriakov J, Ford ME, Duda RL, Jacobs WR Jr, Hendrix RW, Hatfull GF 2000 Genome organization and characterization of mycobacteriophage Bxb1. Mol Microbiol 38:955–970. 11123671 10.1046/j.1365-2958.2000.02183.x

[47] Ghosh P, Kim AI, Hatfull GF 2003 The orientation of mycobacteriophage Bxb1 integration is solely dependent on the central dinucleotide of attP and attB. Mol Cell 12:1101–1111. doi:10.1016/S1097-2765(03)00444-1. 14636570

[48] Ghosh P, Pannunzio NR, Hatfull GF 2005 Synapsis in phage Bxb1 integration: selection mechanism for the correct pair of recombination sites. J Mol Biol 349:331–348. doi:10.1016/j.jmb.2005.03.043. 15890199

[49] Ghosh P, Wasil LR, Hatfull GF 2006 Control of phage Bxb1 excision by a novel recombination directionality factor. PLoS Biol 4:e186. doi:10.1371/journal.pbio.0040186. 16719562 PMC1470463

[50] Savinov A, Pan J, Ghosh P, Hatfull GF 2012 The Bxb1 gp47 recombination directionality factor is required not only for prophage excision, but also for phage DNA replication. Gene 495:42–48. doi:10.1016/j.gene.2011.12.003. 22227494 PMC3273658

[51] Singh S, Ghosh P, Hatfull GF 2013 Attachment site selection and identity in Bxb1 serine integrase-mediated site-specific recombination. PLoS Genet 9:e1003490. doi:10.1371/journal.pgen.1003490. 23658531 PMC3642061

[52] Keenholtz RA, Grindley ND, Hatfull GF, Marko JF 2016 Crossover-site sequence and DNA torsional stress control strand interchanges by the Bxb1 site-specific serine recombinase. Nucleic Acids Res 44:8921–8932. doi:10.1093/nar/gkw724. 27550179 PMC5062993

[53] McGinness KE, Baker TA, Sauer RT 2006 Engineering controllable protein degradation. Mol Cell 22:701–707. 16762842 10.1016/j.molcel.2006.04.027

[54] Kim J-H, O'Brien KM, Sharma R, Boshoff HIM, Rehren G, Chakraborty S, Wallach JB, Monteleone M, Wilson DJ, Aldrich CC, Barry CE, Rhee KY, Ehrt S, Schnappinger D 2013 A genetic strategy to identify targets for the development of drugs that prevent bacterial persistence. Proc Natl Acad Sci U S A 110:19095–19100. doi:10.1073/pnas.1315860110. 24191058 PMC3839782

[55] Klotzsche M, Ehrt S, Schnappinger D 2009 Improved tetracycline repressors for gene silencing in mycobacteria. Nucleic Acids Res 37:1778–1788. doi:10.1093/nar/gkp015. 19174563 PMC2665214

[56] Kim JH, Wei JR, Wallach JB, Robbins RS, Rubin EJ, Schnappinger D 2011 Protein inactivation in mycobacteria by controlled proteolysis and its application to deplete the beta subunit of RNA polymerase. Nucleic Acids Res 39:2210–2220. doi:10.1093/nar/gkq1149. 21075796 PMC3064785

[57] Schnappinger D, Ehrt S 17 1 2014 Regulated expression systems for mycobacteria and their applications. Microbiol Spect doi:10.1128/microbiolspec.MGM2-0018-2013. PMC4254785 25485177

[58] Santi I, Dhar N, Bousbaine D, Wakamoto Y, McKinney JD 2013 Single-cell dynamics of the chromosome replication and cell division cycles in mycobacteria. Nat Commun 4:2470. doi:10.1038/ncomms3470. 24036848

[59] Meniche X, Otten R, Siegrist MS, Baer CE, Murphy KC, Bertozzi CR, Sassetti CM 2014 Subpolar addition of new cell wall is directed by DivIVA in mycobacteria. Proc Natl Acad Sci U S A 111:E3243–E3251. doi:10.1073/pnas.1402158111. 25049412 PMC4128124

[60] Wei JR, Krishnamoorthy V, Murphy K, Kim JH, Schnappinger D, Alber T, Sassetti CM, Rhee KY, Rubin EJ 2011 Depletion of antibiotic targets has widely varying effects on growth. Proc Natl Acad Sci U S A 108:4176–4181. doi:10.1073/pnas.1018301108. 21368134 PMC3053961

[61] Marinelli LJ, Piuri M, Swigoňová Z, Balachandran A, Oldfield LM, van Kessel JC, Hatfull GF 2008 BRED: a simple and powerful tool for constructing mutant and recombinant bacteriophage genomes. PLoS One 3:e3957. doi:10.1371/journal.pone.0003957. 19088849 PMC2597740

[62] Bardarov S, Bardarov S Jr, Pavelka MS Jr, Vasan Sambandamurthy V, Larsen M, Tufariello J, Chan J, Hatfull G, Jacobs WR Jr. 2002 Specialized transduction: an efficient method for generating marked and unmarked targeted gene disruptions in *Mycobacterium tuberculosis*, *M. bovis* BCG and *M. smegmatis*. Microbiology 148:3007–3017. doi:10.1099/00221287-148-10-3007. 12368434

[63] Wang X, Tang B, Ye Y, Mao Y, Lei X, Zhao G, Ding X 2017 Bxb1 integrase serves as a highly efficient DNA recombinase in rapid metabolite pathway assembly. Acta Biochim Biophys Sin 49:44–50. doi:10.1093/abbs/gmw115. 27864282

[64] Xu Z, Thomas L, Davies B, Chalmers R, Smith M, Brown W 2013 Accuracy and efficiency define Bxb1 integrase as the best of fifteen candidate serine recombinases for the integration of DNA into the human genome. BMC Biotechnol 13:87. doi:10.1186/1472-6750-13-87. 24139482 PMC4015280

[65] Datta S, Costantino N, Zhou X, Court DL 2008 Identification and analysis of recombineering functions from Gram-negative and Gram-positive bacteria and their phages. Proc Natl Acad Sci U S A 105:1626–1631. doi:10.1073/pnas.0709089105. 18230724 PMC2234195

[66] Kaps I, Ehrt S, Seeber S, Schnappinger D, Martin C, Riley LW, Niederweis M 2001 Energy transfer between fluorescent proteins using a co-expression system in *Mycobacterium smegmatis*. Gene 278:115–124. doi:10.1016/S0378-1119(01)00712-0. 11707328

[67] Schindelin J, Arganda-Carreras I, Frise E, Kaynig V, Longair M, Pietzsch T, Preibisch S, Rueden C, Saalfeld S, Schmid B, Tinevez J-Y, White DJ, Hartenstein V, Eliceiri K, Tomancak P, Cardona A 2012 Fiji: an open-source platform for biological-image analysis. Nat Methods 9:676–682. doi:10.1038/nmeth.2019. 22743772 PMC3855844

