# Supplementary figures and images for: ORBIT: a New Paradigm for Genetic Engineering of Mycobacterial Chromosomes
Source: mBio. 2018 Dec 11;9(6):e01467-18. doi: 10.1128/mBio.01467-18 (PMC6299477; doi:10.1128/mBio.01467-18)

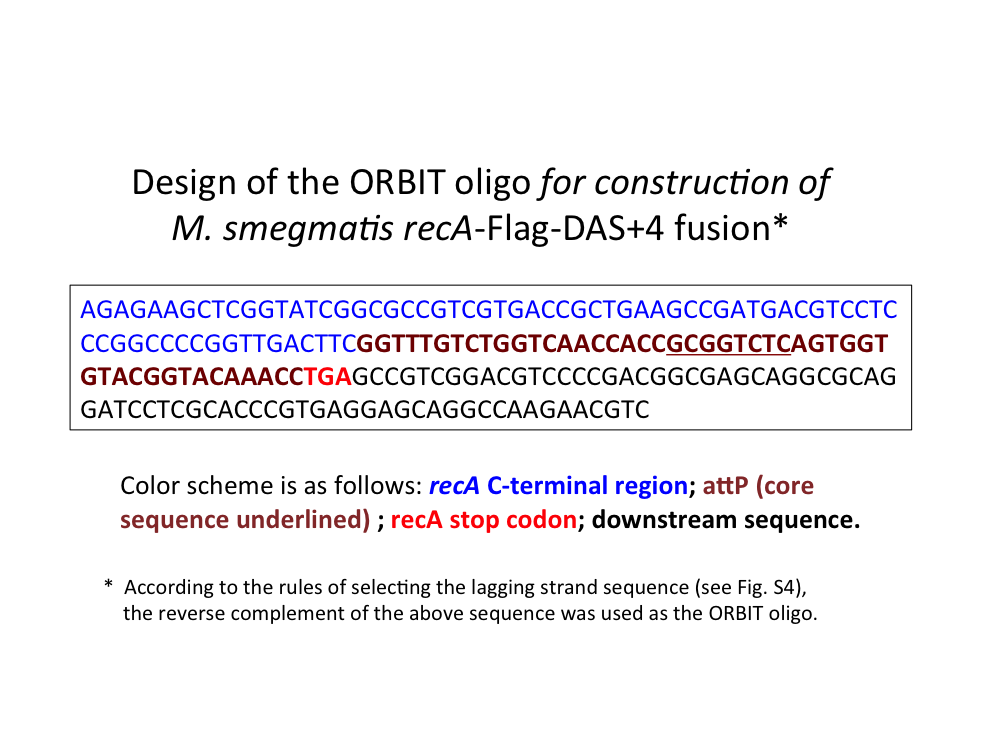

Supplement: FIG S1 [file mbo006184224sf1.tif]

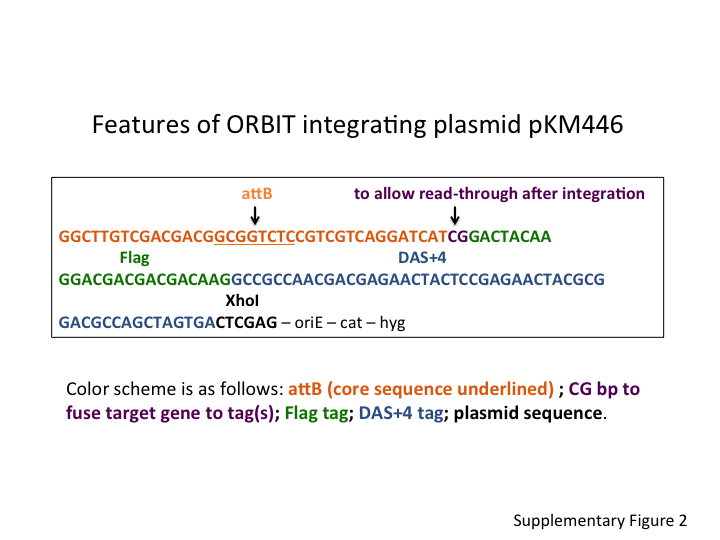

Supplement: FIG S2 [file mbo006184224sf2.tif]

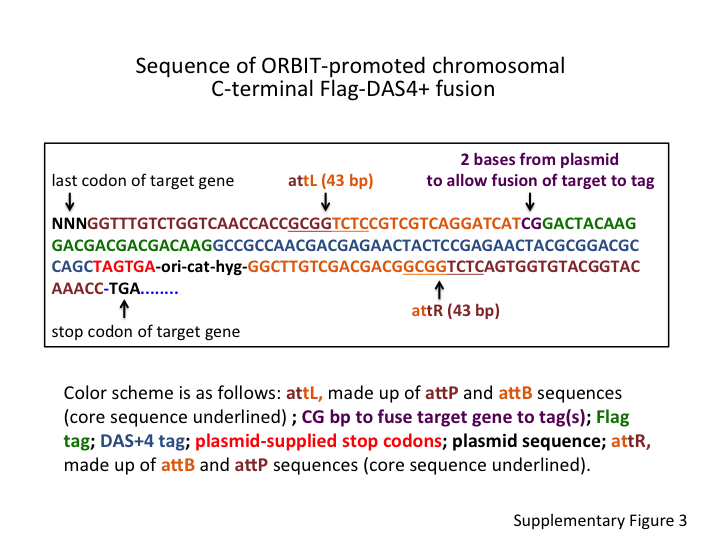

Supplement: FIG S3 [file mbo006184224sf3.tif]

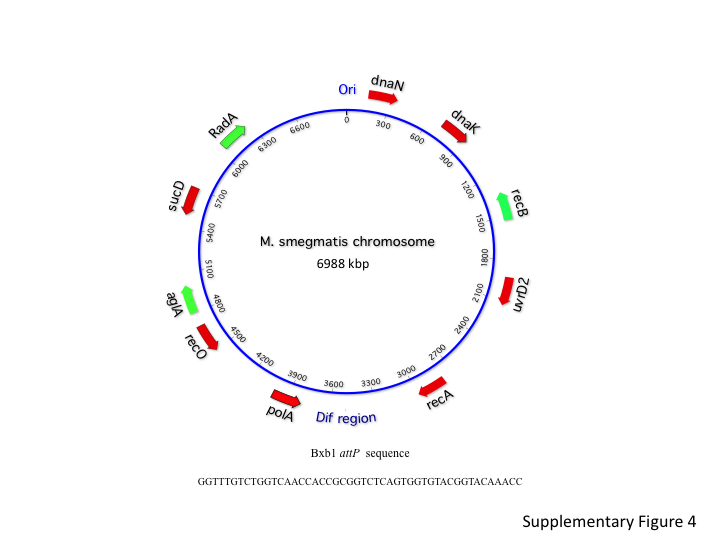

Supplement: FIG S4 [file mbo006184224sf4.tif]
